# Ribosomal protein RPL5 regulates colon cancer cell proliferation and migration through MAPK/ERK signaling pathway

**DOI:** 10.1186/s12860-022-00448-z

**Published:** 2022-11-16

**Authors:** Huahua Zhang, Junli Liu, Qingqing Dang, Xueru Wang, Jie Chen, Xiaoyin Lin, Na Yang, Juan Du, Haiyan Shi, Yong Liu, Jiming Han

**Affiliations:** 1 Medical Research and Experimental Center, Medical College, Yan’an University, Yan’an, 716000 China; 2 Surgical anesthesiology, Yan’an People’s Hospital, Yan’an, 716000 China; 3 Cardiovascular Surgery, the Second Affiliated Hospital of Xi’an Jiaotong University, Xi’an, 710004 China; 4 General Surgery, Yan’an People’s Hospital, Yan’an, 716000 China

**Keywords:** Ribosomal protein RPL5, Colon cancer, Proliferation, Migration, MAPK/ERK signaling pathway

## Abstract

**Background:**

Abnormal expression of ribosomal proteins has an important regulatory effect on the progression of cancer. RPL5 is involved in the progression of various malignancies, however, the role of RPL5 in colon cancer remains is still unclear.

**Methods:**

Data from TCGA and GTEx databases were used to analyze the RPL5 expression in pan-cancer. The expression level of RPL5 in clinical colon cancer tissue samples and human colon cancer cell lines was detected by western blotting; siRNA targeting RPL5 was designed, and its interference efficiency was verified by western blotting and RT-qPCR; CCK8 assay, clone formation assay, cell cycle assay, and cell scratch assay were used to observe the effect of RPL5 on colon cancer cell proliferation and migration; the changes of proteins related to MAPK/ERK signaling pathway were also detected using western blotting.

**Results:**

The expression level of RPL5 in colon cancer tissues and cell lines was significantly higher than that in adjacent tissues and NCM460 cells, respectively, and its expression level was higher in HCT116 cells and RKO cells. Knockdown of RPL5 significantly inhibited the proliferation and migration of HCT16 and RKO cells, and arrested the cell cycle in G0/G1 phase. Mechanistic studies revealed that the expression of p-MEK1/2, p-ERK, c-Myc were down-regulated, and the expression of FOXO3 was up-regulated after down-regulation of RPL5, ERK activator (TBHQ) could partially reverse the above-mentioned effects caused by siRPL5. Moreover, TBHQ could partially reverse the inhibitory effect of siRPL5 on the proliferation and migration of colon cancer cells. Collectively, RPL5 promoted colon cell proliferation and migration, at least in part, by activating the MAPK/ERK signaling pathway.

**Conclusion:**

RPL5 promoted colon cell proliferation and migration, at least in part, by activating the MAPK/ERK signaling pathway, which may serve as a novel therapeutic target for cancers in which MAPK/ERK signaling is a dominant feature.

**Supplementary Information:**

The online version contains supplementary material available at 10.1186/s12860-022-00448-z.

## Introduction

Colorectal cancer (CRC) currently ranks third in the global incidence spectrum and second cause in the number of deaths [1]. The incidence of CRC is uniquely increasing in young adults in nine high-income countries across North America, Europe and Oceania [2]. Although recent advances in surgical resection techniques have improved survival in patients with early CRC, the long-term prognosis for most patients with CRC remains poor, largely due to recurrence and metastasis [3, 4].. Currently, the exact mechanisms underlying CRC development remain unknown. Therefore, identifying key molecules involved in CRC progression may help provide new ideas for clinical treatment of CRC with a view to improving the prognosis of CRC patients.

Ribosomal protein (RP) is a widely existing RNA-binding protein that was previously thought to function only as a combination of ribosomal RNA (rRNA) involved in protein synthesis. More and more studies have found that abnormal expression or mutation of ribosomal proteins has an important regulatory effect on the occurrence and development of cancer [5]. The ribosomal protein RPL5 is a component of the ribosome, a large ribonucleoprotein complex responsible for protein synthesis in cells. RPL5 is involved in the development and progression of a variety of tumors, such as melanoma [6], liver cancer [7], breast cancer [8], and non-small cell lung cancer [9]. However, the role and regulation mechanism of RPL5 has not been revealed in colon cancer.

This study examines the effect of RPL5 on the progression of colon adenocarcinoma (COAD), including cell proliferation, migration and determines its underlying molecular mechanism using CRC cell lines in vitro. Here, we demonstrate that RPL5 promotes COAD cell proliferation and migration via the MAPK/ERK signaling pathway. These findings suggest that RPL5 plays an important role in regulating COAD progression and may serve as a prognostic biomarker for COAD.

## Materials and methods

### RPL5 expression in normal tissues and pan-cancer

The levels of RPL5 mRNA (transcript RNA-seq datasets) across the pan-cancer were analyzed using data obtained TCGA from UCSC Xena (https://xena.ucsc.edu). Considering the small number of normal samples in TCGA, we combined the normal tissue data from GTEx database with the TCGA tumor tissue data to investigate the differential expression of RPL5 in 33 cancerous tissues and adjacent tissues.

### RPL5 expression and its relationship with clinical stage of COAD

Based on the above pan-cancer analysis, we further focused on the impact of RPL5 expression on the clinical stage of COAD patients in the database cohort. Data on COAD cases and normal controls were downloaded from the UC Santa Cruz website (https://xena.ucsc.edu). Therefore, tumor samples were matched to clinical stage to obtain data on RPL5 expression and clinical stage, and their relationship was analyzed.

### Clinical samples

Formalin-fixed paraffin-embedded tissue blocks of COAD tumors and adjacent normal mucosal tissues were obtained from 11 patients between March 2021 and November 2021 at the Yan’an People’s Hospital (Yan’an, China). None of the patients received prior chemotherapy, radiotherapy or systemic therapies, or had additional malignant tumors. The study protocol was approved by Medical School of Yan’an University’ Ethics Committee (NO.2022057), with written informed consent of all patients.

### Cell culture and siRNA transfection

Human colon cancer cell lines (HCT116 and RKO) were gifted by the Key Laboratory of Environment and Genes Related to Diseases of Xi’an Jiaotong University. RKO cells were maintained in DMEM medium (Biological Industries, Beit Haemek, Israel) supplemented with 10% fetal bovine serum (Biological Industries, Beit Haemek, Israel). HCT116 cells were maintained in RPMI-1640 medium (Biological Industries, Beit Haemek, Israel) supplemented with 10% fetal bovine serum (Biological Industries, Beit Haemek, Israel). All cells were cultured in a humidified 5% CO_2_ incubator at 37 °C. Human RPL5 siRNAs (siRPL5–1 sense, 5′-GGGAGCUGUGGAUGGAGGCTT-3′; siRPL5–1 antisense, 5′-GCCUCCAUCCACAGCUCCCTT-3′; siRPL5–2 sense, 5′-CUGCCAAAAUAUGGUGUGATT-3′; siRPL5–2 antisense, 5′-UCACACCAUAUUUUGGCAGTT-3′;) and negative control siRNA (siNC sense, 5′- UUCUCCGAACGUGUCACGU-3′; and siNC antisense, 5′-ACGUGACACGUUCGGAGAA-3′) were chemically synthesized by GenePharma (Shanghai, China). Transfection of siRNAs was performed using jetPRIME reagent (Polyplus-transfection SA), according to the manufacturer’s protocol.

### Western blotting

Cells were lysed in RIPA buffer (Pioneer, Shanghai, China) supplemented with protease inhibitor cocktail and phosphatase inhibitor cocktail (TargetMol, USA). Protein concentration determined using a BCA protein assay kit (Proteintech, Wuhan, China). The protein samples were transferred to PVDF membrane by SDS-PAEG, membranes were incubated with specific primary antibody at room temperature for 30 min and then overnight at 4 °C. Then they were incubated with corresponding anti-rabbit/anti-mouse secondary antibody (TransGen Biotech, Beijing) at room temperature for 1 h. The membranes were incubated with ECL (Boster Biological Technology co.ltd, California, USA) in the dark for chemiluminescence detection. Luminescent signals were detected and recorded by Syngene GBox (Syngene, Cambridge, UK). The primary and secondary antibodies used are listed as follows: RPL5 (Proteintech, 29,092–1-AP), MMP2 (Proteintech, 10,373–2-AP), MMP9 (Proteintech, 10,375–2-AP), CDK4 (Proteintech, 11,026–1-AP), CyclinD1 (Proteintech, 26,939–1-AP), MEK1/2 (Proteintech, 11,049–1-AP), p-ERK (Proteintech, 28,733–1-AP), ERK (Proteintech, 67,170–1-Ig), c-Myc (Proteintech, 10,828–1-AP), p-MEK1/2 (Cell Signaling Technology, 9154), FOXO3 (Boster, BM4734) and β-Tubulin (Proteintech, 66,240–1-Ig).

### RNA isolation and RT-qPCR

Total RNA from the human CRC cells was extracted using TRIzol reagent (Invitrogen; Thermo Fisher Scientific, Inc.) according to the manufacturer’s instructions. mRNA was reverse transcribed into cDNA using *EasyScript* One-Step gDNA Removal and cDNA Synthesis SuperMix (TransGen Biotech, Beijing) according to the manufactures instruction. The primers were synthesized by Tsingke Biotech, and the sequences were as follows: RPL5 forward primer-GCTTATGCCCGTATAGAGGGG，RPL5 reverse primer-GCCAAACCTATTGAGAAGCCTG; GAPDH forward primer-GGAGCGAGATCCCTCCAAAAT, GAPDH reverse primer -GGCTGTTGTCATACTTCTCATGG. qPCR was performed using *PerfectStart*Green qPCR SuperMix (TransGen Biotech, Beijing) on a Cobas z480 instrument, according to the manufacturer’s instructions. The relative expression of RPL5 to β-actin (internal control) was calculated using the 2^-ΔΔCt^ method [10].

### CCK-8 assay

In brief, colon carcinoma cells HCT116 and RKO (3 × 10^3^/cells per well) were seeded in 96-well culture plates. About 10 μl of CCK8 (TargetMol, USA) solution was added to the 96-well at 24, 48 and 72 h after transfection and cells were incubated in 37 °C for 2 h. The absorbance was measured on a microplate reader (MD, USA) at 450 nm.

### Clone formation assay

Transfected HCT116 and RKO cells were plated in 6-well plates at a density of 800 cells in triplicate and incubated at 37 °C with 5% CO_2_ for 7–10 days until they reached 80% confluence. Cells were then fixed with 4% paraformaldehyde and stained with 0.5% crystal violet (Sigma Aldrich; Merck KGaA) for 30 min at room temperature. Images were captured on a G: BOX XR5 Gel Imaging System (Syngene, UK). After dissolving in DMSO, colonies were quantified and the absorbance was measured on a microplate reader (MD, USA) at 570 nm.

### Wound healing assay

Wound healing assay was performed to measure cell migration capacity. Briefly, once cells had grown to 80–90% confluence in 6-well plates, a single scratch wound was generated with a 10 μl disposable pipette tip. Images of the cells were captured at 40× magnification (Nikon, Tokyo, Japan) at 0, 24, 48 and 72 h and used to determine cell migration.

### Cell cycle assay

HCT116 and RKO cells were seeded in 6-well plates (30 × 10^4^/cells per well). 24 h after transfection, cells were collected by trypsinization, washed with PBS and fixed ice-cold 70% ethanol at 4 °C overnight. Then cells were washed with PBS twice. And the cells were incubated with 150 μl RNase for 15 min at room temperature and stained with 150 μl propidium iodide for 30 min at 4 °C in the dark. Cell cycle distributions were measured using a flow cytometer (Syngene, USA).

### TBHQ treatment

Tert-Butylhydroquinone (TBHQ; HY-100,489) purchased from MedChemExpress (Shanghai, China) and was dissolved in DMSO. For western blotting, TBHQ was added 6 h after transfection and incubated for 48 h. For CCK8 assay and cell scratch assay, TBHQ was added 6 h after transfection and the cell viability and cell migration ability were detected by incubating for 24 h and 48 h respectively.

### Statistical analysis

All experiments were performed with three independent replicates. The data were expressed as the mean ± standard deviation and analyzed with SPSS 22.0 software (SPSS, Inc.). Student’s t-test was used for comparison of two groups and the one-way analysis of variance (ANOVA) followed was performed for multi-group comparison *P* < 0.05 was considered to be statistically significant.

## Results

### RPL5 expression in normal tissues and pan-cancer

**Fig. 1:**
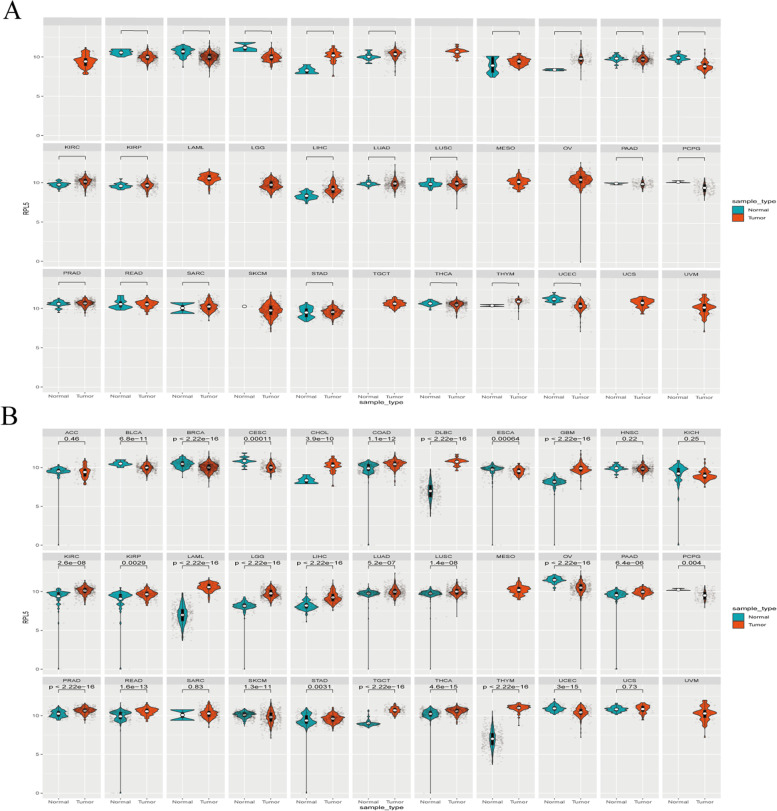
RPL5 expression in pan-cancer. **A** The expression of RPL5 in different human tumors within TCGA. **B** RPL5 expression in 33 human tumors was analyzed by integrating normal tissue data from GTEx database and TCGA tumor tissue. (TCGA sample data and GTEx normal sample data of 33 tumors) (blue: normal; red: tumor; log2(TPM + 1), * *P* < 0.05, ** *P* < 0.01, *** *P* < 0.001)

Numerous studies have reported that RPL5 was involved in the progression of various malignant tumors [6–9]. The pan-cancer gene expression patterns were performed to examine the differential expression of RPL5 across TCGA cancer types. The results showed that the expression level of RPL5 was up-regulated in tissues of most cancer types, including COAD, compared with corresponding adjacent tissues (Fig. 1A). Considering the small number of normal samples in TCGA, and to extend this comparison, we combined normal tissue data from the GTEx database and TCGA tumor tissue data to analyze the expression of 33 tumors and obtained consistent results: RPL5 expression was elevated in most tumors, including COAD (Fig. 1B).

### Up-regulated expression of RPL5 in COAD

**Fig. 2:**
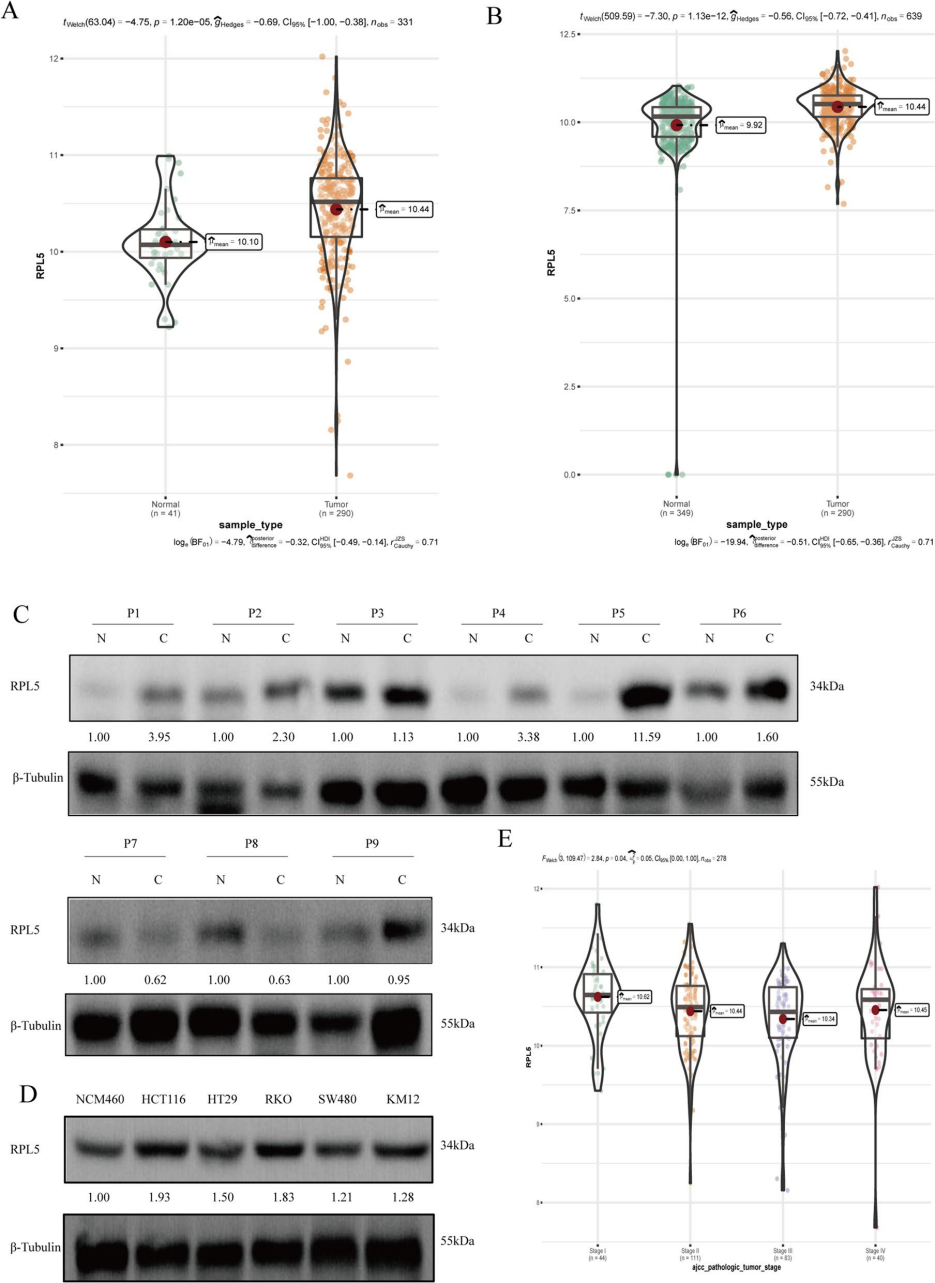
Up-regulated expression of RPL5 in COAD. **A** Data from TCGA database demonstrated a significant upregulation of RPL5 expression in COAD tumor samples (*n* = 290) compared with normal tissue (*n* = 41). **B** Data from GTEx database and TCGA tumor tissue demonstrated a significant upregulation of RPL5 expression in COAD tumor samples (*n* = 290) compared with normal tissue (*n* = 349). **C** Nine pairs of cancer tissues and paired adjacent tissues from clinical colon cancer patients were collected, and the differential expression of RPL5 in colon cancer tissues and paired adjacent tissues was detected by western blotting. The blots were cut prior to hybridization with antibodies during blotting. **D** The proteins of human normal colon epithelial cells NCM460 and colon cancer cells HCT116, HT29, RKO, SW480 and KM12 were extracted, and the expression of RPL5 in colon cancer was detected by western blotting. The blots were cut prior to hybridization with antibodies during blotting. **E** The TCGA database was used to analyze the correlation between the expression of RPL5 and the stage of clinical colon cancer patients

Through the analysis of normal tissue data in the TCGA database and tumor tissue data in the GTEx database and TCGGA database, it was found that RPL5 was highly expressed in COAD (Fig. 2A, B). The expression of RPL5 in clinical colon cancer tissues was detected by western blotting, and the results showed that compared with adjacent tissues, the expression of RPL5 in colon cancer tissues increased (Fig. 2C). Compared with normal colon epithelial cell lines NCM460, the protein levels of RPL5 were increased in colon cancer cell lines (Fig. 2D). The TCGA database was used to analyze the correlation between the expression of RPL5 and the stage of clinical colon cancer patients, and the results showed that the expression of RPL5 was correlated with the stage of the patients (Fig. 2E).

### RPL5 expression is decreased by siRPL5 transfection in human colon cancer cells

**Fig. 3:**
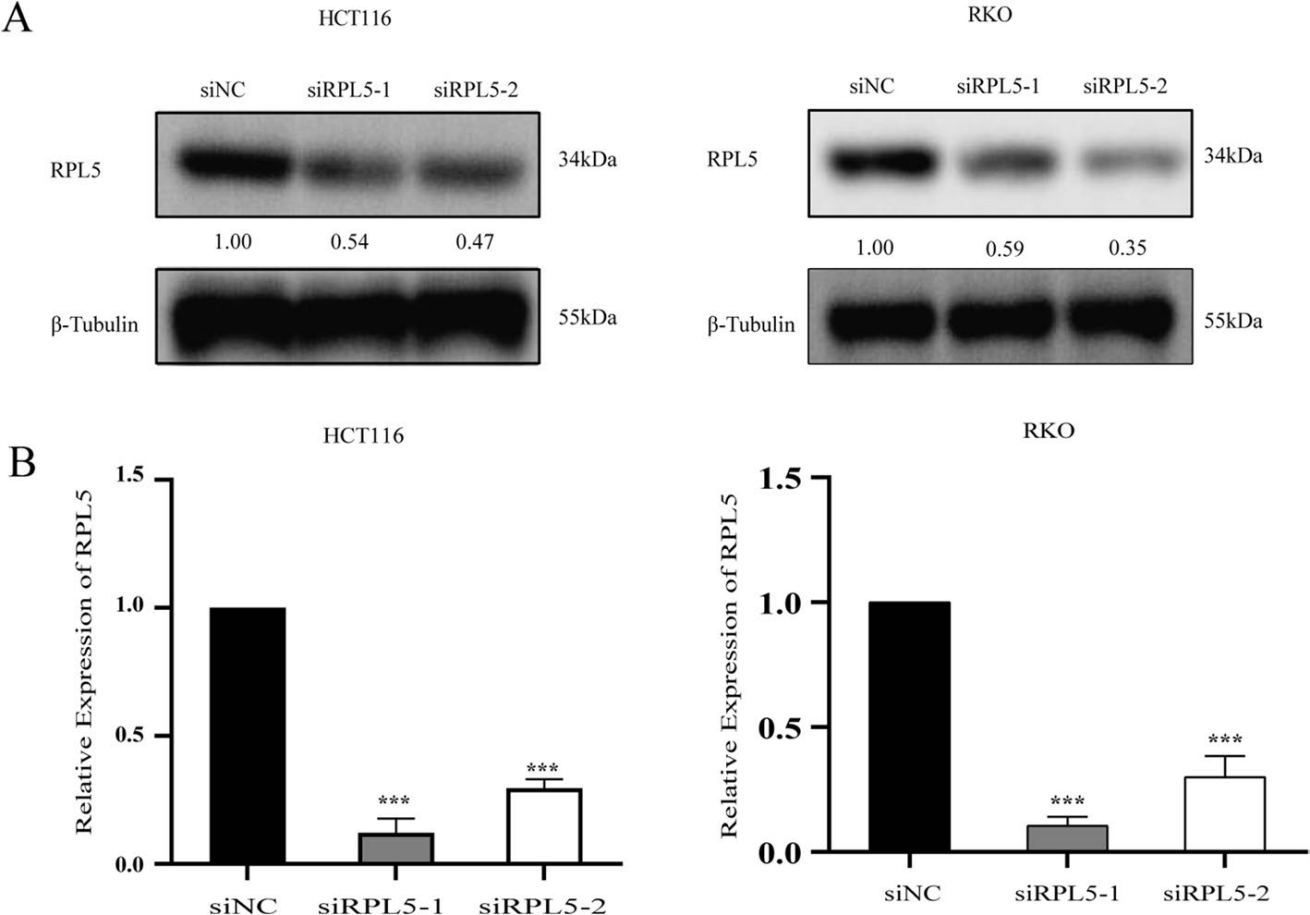
RPL5 expression is decreased by siRPL5 transfection in human colon cancer cells. **A** Western blotting and (**B**) reverse transcription-qPCR were performed and revealed a significant decrease in the protein and mRNA levels of RPL5 in siRPL5–1- and siRPL5–2-transfected human colon cancer cells, compared with those observed in siNC-transfected cells. The band intensities of RPL5 protein were quantified relative to β-Tubulin and normalized to the siNC sample. The blots were cut prior to hybridization with antibodies during blotting. The experiments were repeated three independent times with reproducible results. (***P < 0.001)

To further confirm the role of RPL5 in colon cancer, we designed interference fragments targeting RPL5 (siRPL5) and control (siNC), and western blotting and RT-qPCR were used to verify the transfection efficiency of HCT116 and RKO cells. As an obvious result, compared with siNC transfection group, the protein level and mRNA level of siRPL5 transfection group were significantly decreased (Fig. 3A, B).

### The knockdown of RPL5 expression levels inhibits the proliferation capability

**Fig. 4:**
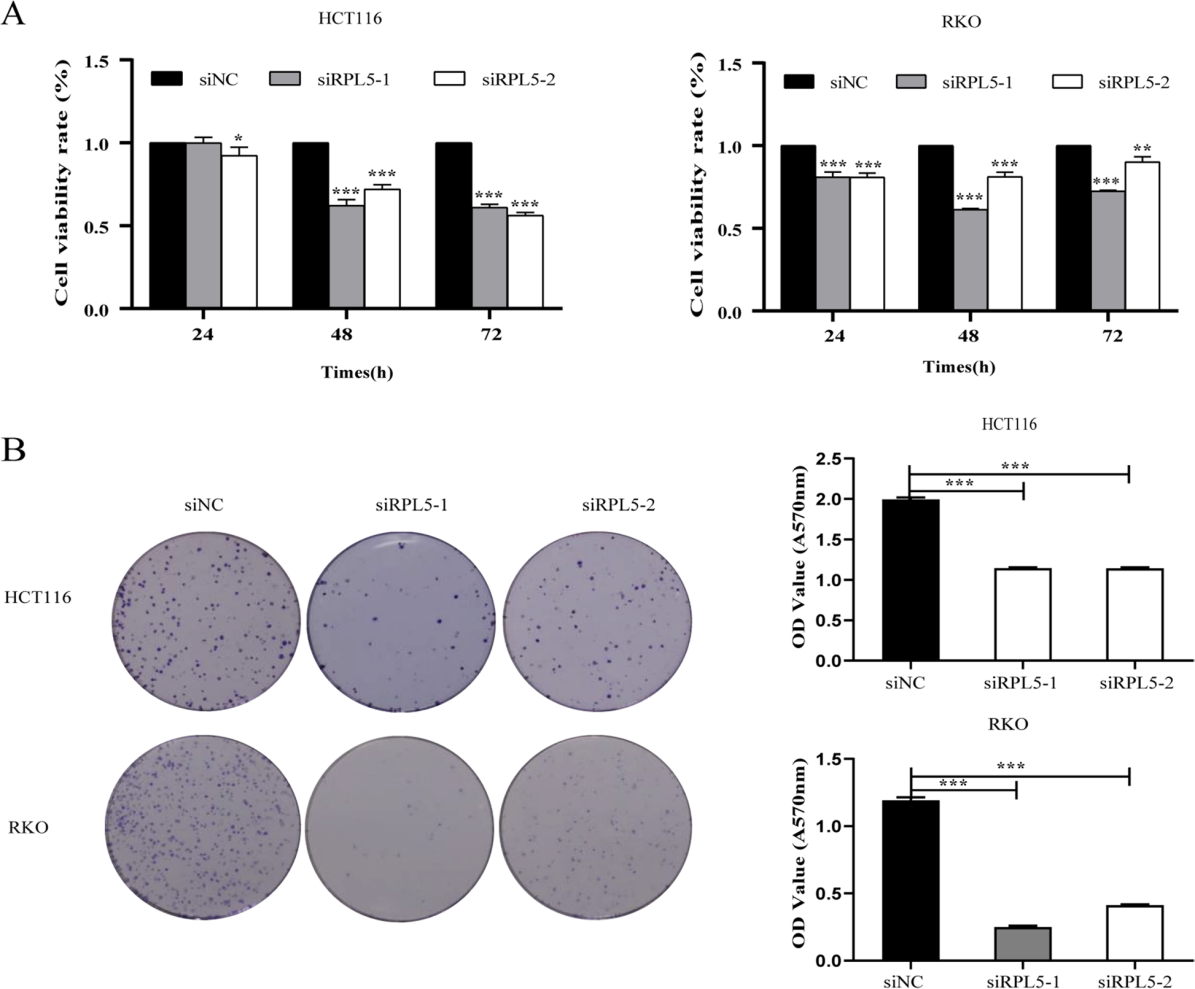
The knockdown of RPL5 expression levels inhibits the proliferation capability. **A** RPL5 knockdown significantly inhibited the proliferation of HCT116 and RKO cells using CCK-8 assay. **B** Cell clone formation assay demonstrated that knockdown of RPL5 reduced the clone formation ability of HCT116 and RKO cells. (*P < 0.05, ** *P* < 0.01, *** *P* < 0.001)

The effects of RPL5 on the proliferation of colon cancer cell lines HCT116 and RKO were investigated by CCK-8 assay and cell clone formation assay. Firstly, compared with the siNC transfection group, it was found that siRPL5 significantly inhibited the proliferation ability of HCT116 and RKO using CCK-8 assay (Fig. 4A). The results of cell cloning formation assay displayed that compared with the siNC transfection group, the cloning formation ability of colon cancer cells transfected with siRPL5 was weakened (Fig. 4B). These results indicate that the knockdown of RPL5 expression levels inhibits the proliferation capability.

### Knockdown of RPL5 inhibits the migration capability of human colon cancer cells

**Fig. 5:**
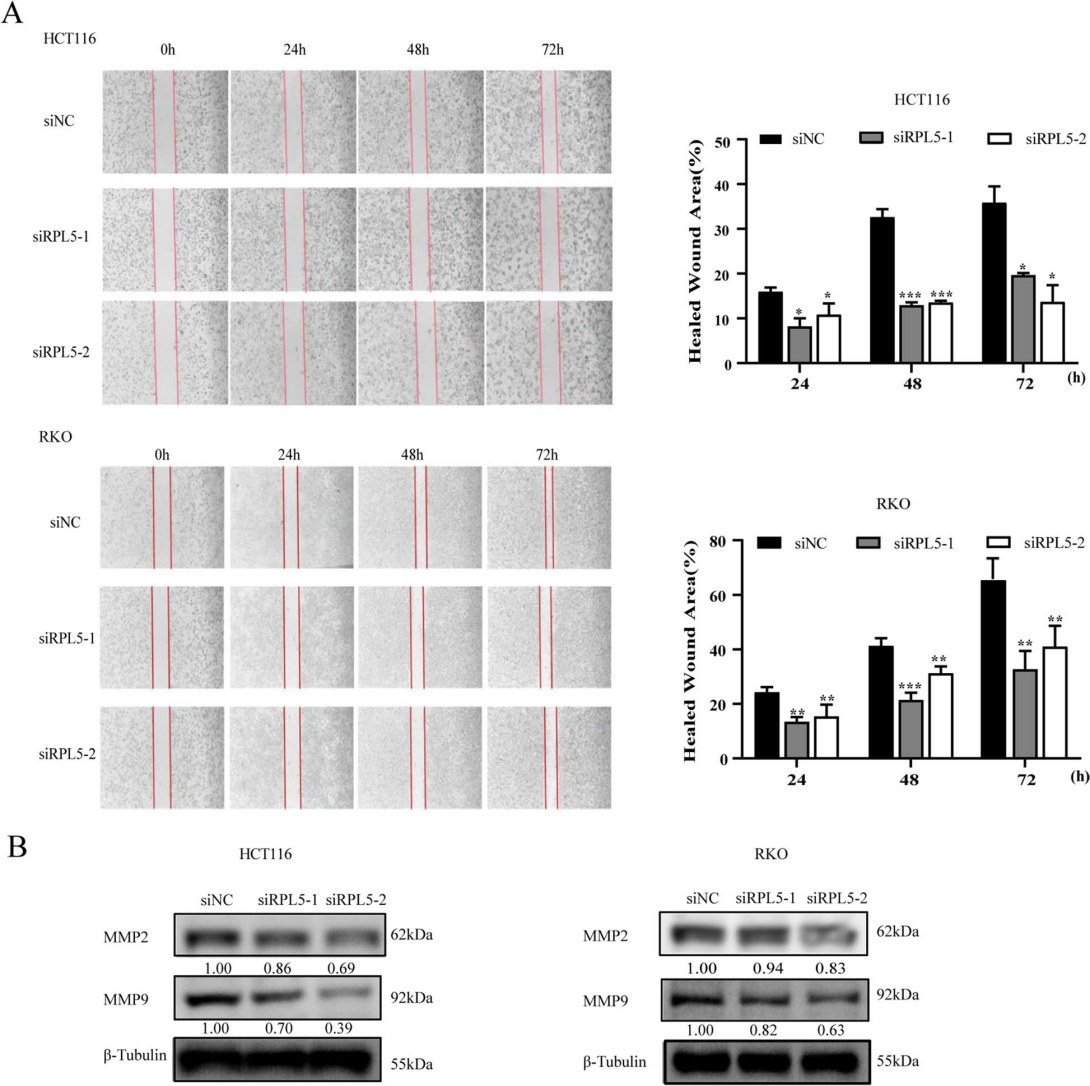
Knockdown of RPL5 inhibits the migration capability of human colon cancer cells. **A** Wound healing assay demonstrated a significant decrease in the number of migration cells in siRPL5–1- and siRPL5–2-transfected human colon cancer cells compared with that observed in siNC-transfected cells. Scale bar, 100 μm. **B** The levels of MMP2 and MMP9 were detected using western blotting. The band intensities of MMP2 and MMP9 protein were quantified relative to β-Tubulin and normalized to the siNC sample. The blots were cut prior to hybridization with antibodies during blotting. All experiments were repeated three independent times with reproducible results. (*P < 0.05, ** *P* < 0.01, *** *P* < 0.001)

In order to further observe the effect of RPL5 on the migration ability of colon cancer cells, wound healing assay was performed. The results showed that compared with the siNC transfection group, the migration ability of colon cancer cells in the siRPL5 transfected group was reduced (Fig. 5A). Meanwhile, MMP2 and MMP9 expression in siRPL5-transfected colon cancer cells was lower than that of siNC transfection group (Fig. 5B). These results suggest that knockdown of RPL5 inhibits the migration capability of human colon cancer cells.

### The knockdown of RPL5 arrests the colon cancer cell cycle in G0/G1 phase

**Fig. 6:**
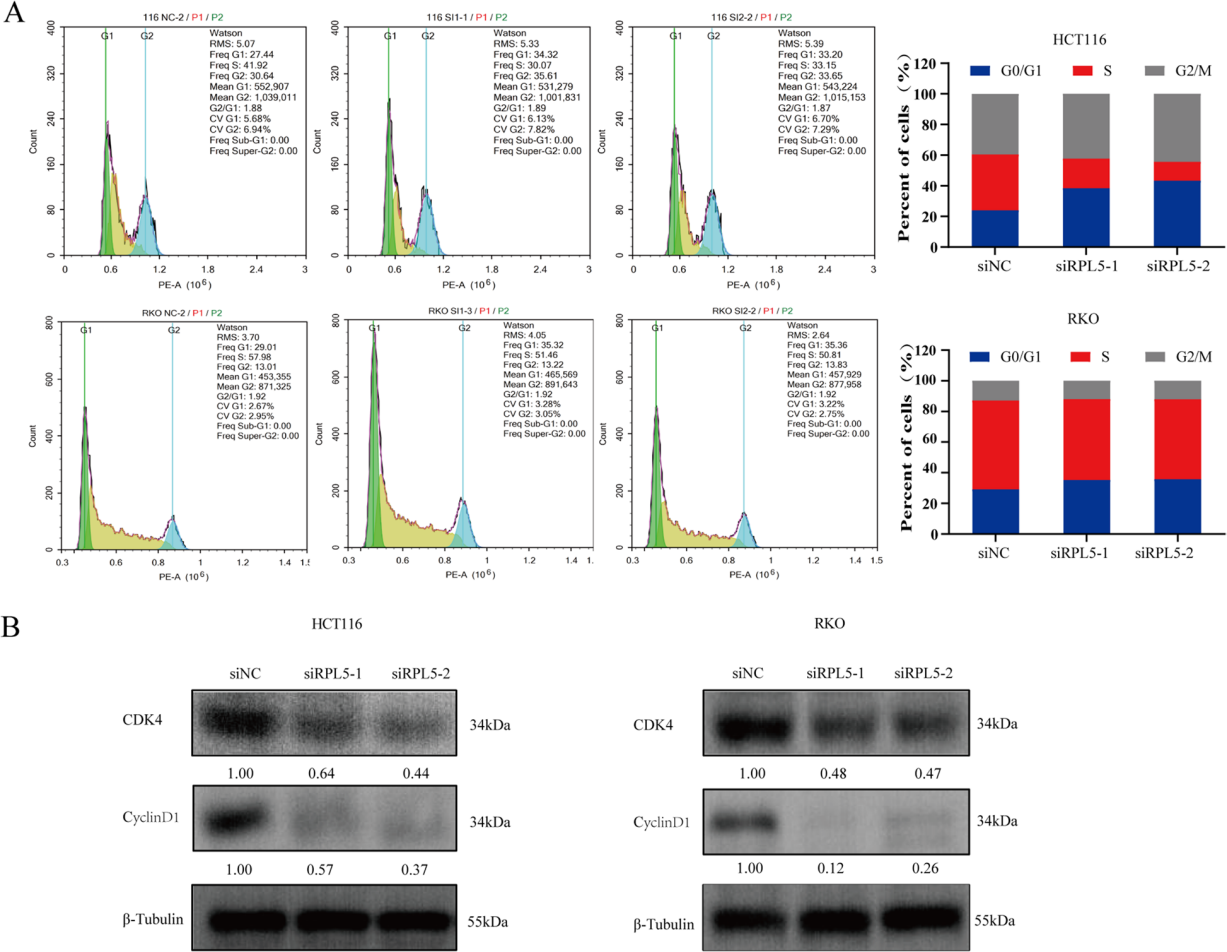
The knockdown of RPL5 arrests the colon cancer cell cycle in G0/G1 phase. **A** Flow cytometry showed that knockdown of RPL5 increased the proportion of colon cancer cells in G0/G1 phase. **B** The levels of G0/G1 phase related proteins CDK4 and CyclinD1 were detected by western blotting. The band intensities of CDK4 and CyclinD1 protein were quantified relative to β-Tubulin and normalized to the siNC sample. The blots were cut prior to hybridization with antibodies during blotting. The data are representative of three independent experiments

The effect of RPL5 on the cell cycle of colon cancer cells was observed by flow cytometry, and it was found that compared with the siNC-transfected group, the cell cycle was arrested in the G0/G1 phase after siRPL5 transfection (Fig. 6A). Western blotting was used to detect the expression of G0/G1 phase-related proteins in the experimental system. The results showed that compared with the siNC transfection group, the expression levels of G0/G1-related proteins CDK4 and CyclinD1 in the transfection siRPL5 group were reduced (Fig. 6B). Combined with the results of CCK8, it can be seen that siRPL5 blocks the cell cycle of colon cancer cells in G0/G1 phase and thus inhibits their proliferation.

### RPL5 regulates colon cancer cell proliferation and migration through MAPK/ERK signaling pathway

**Fig. 7:**
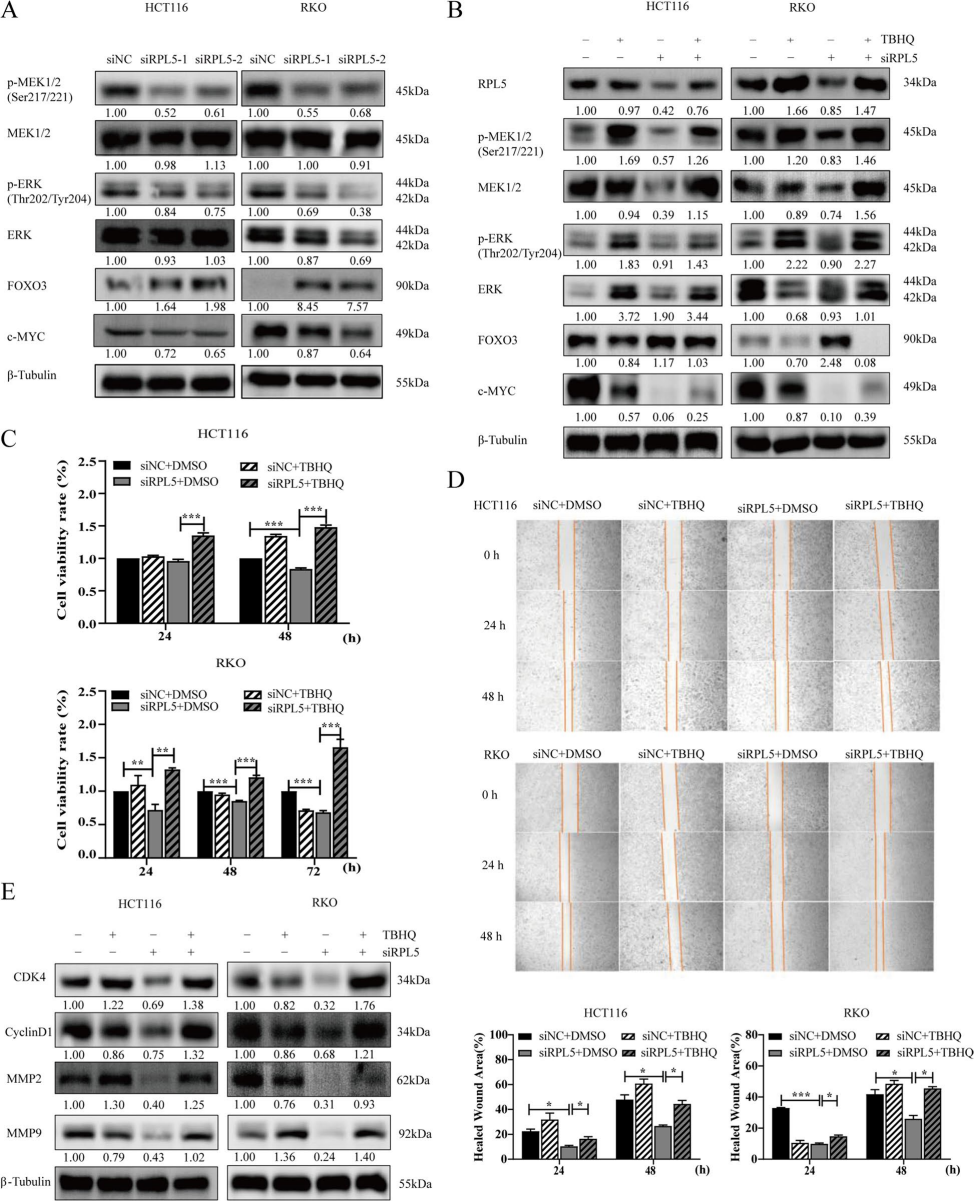
RPL5 regulates colon cancer cell proliferation and migration through MAPK/ERK signaling pathway. **A** HCT116 and RKO cells were treated with RPL5-targeting siRNAs (siRPL5–1 and siRPL5–2) or negative control siRNA (siNC). After 48 h, cell lysates were harvested, and the protein samples were separated by SDS-PAGE. The levels of MAPK/ERK signaling pathway related proteins P-MEK1/2, MEK1/2, P-ERK, ERK, FOXO3 and c-Myc were detected by western blotting. The band intensities of P-MEK1/2, MEK1/2, P-ERK, ERK, FOXO3 and c-Myc proteins were quantified relative to β-Tubulin and normalized to the siNC sample. The blots were cut prior to hybridization with antibodies during blotting. **B**TBHQ (50 μM) was added to HCT116 and RKO cells after 6 h transfection of siRPL5, and the proteins of each group were extracted 48 h later. Changes of proteins related to MAPK/ERK signaling pathway were detected by western blotting. The blots were cut prior to hybridization with antibodies during blotting. **C** HCT116 and RKO cells were transfected with siRPL5 and then added TBHQ (20 μM) to detect the effect of TBHQ on the proliferation inhibition of colon cancer cells caused by siRPL5 at 24 h and 48 h. **D** HCT116 and RKO cells were transfected with siRPL5 and then added TBHQ (20uM) to detect the effect of TBHQ on the inhibition of colon cancer cell migration caused by siRPL5 at 24 h and 48 h. (E) TBHQ (50 μM) was added to HCT116 and RKO cells after transfection of siRPL5 for 6 h, and cell cycle-related proteins (CDK4 and CyclinD1) and migration-related proteins (MMP2 and MMP9) were detected by western blotting. The band intensities of CDK4, CyclinD1, MMP2 and MMP9 proteins were quantified relative to β-Tubulin and normalized to the siNC sample. The blots were cut prior to hybridization with antibodies during blotting. The data are representative of three independent experiments. (*P < 0.05, ** *P* < 0.01, *** *P* < 0.001)

The MAPK/ERK pathway is a key signaling pathway involved in the development of various cancers including CRC [11, 12]. In order to explore the potential mechanism of RPL5 regulation of colon cancer cell proliferation and migration, this study firstly detected the changes of MAPK/ERK signaling pathway-related proteins in the siRPL5 transfection experimental system by western blotting. The experimental results showed that compared with the siNC transfection group, the protein levels of P-MEK and P-ERK in cells transfected with siRPL5 were significantly reduced, and the expression of the downstream target gene c-Myc was down-regulated and the expression of FOXO3 was up-regulated (Fig. 7A). After further treatment with the ERK activator TBHQ, we found that TBHQ could partially reverse the down-regulation of P-MEK, P-ERK and c-Myc expression and the up-regulation of FOXO3 expression caused by siRPL5 (Fig. 7B). To further confirm that RPL5 regulates colon cancer cell proliferation and migration through MAPK/ERK signaling pathway, we observed whether TBHQ could reverse or partially reverse the inhibitory effect of siRPL5 on colon cancer cell proliferation and migration by CCK8 assay and wound healing assay. The experimental results indicated that TBHQ could partially reverse the inhibitory effect of siRPL5 on the proliferation and migration of colon cancer cells (Fig. 7C, D). Based on the previous results (Figs. 5, 6), we detected the changes in cell cycle-related proteins (CDK4, CyclinD1) and cell migration-related proteins (MMP2, MMP9) by western blotting, and the results showed that TBHQ can partially reverse the decrease in the expression of CDK4, CyclinD1, MMP2, MMP9 proteins caused by siRPL5(Fig. 7E). In summary，RPL5 regulates colon cancer cell proliferation and migration through MAPK/ERK signaling pathway.

## Discussion

As part of the 5S RNP/5S ribonucleoprotein particle, RPL5 is an important component of the large subunit and requires for rRNA formation and maturation [13, 14]. In recent years, studies have shown that in addition to participating in the assembly function of basic translation machinery, ribosomal proteins also exert their extra-ribosome functions in cells, including regulating gene expression, regulating cell cycle, and regulating cell proliferation and apoptosis [15]. Numerous studies have reported that RPL5 is involved in the progression of various malignant tumors. The study by Yumei Li et al. found that WDR74 regulates the occurrence and metastasis of melanoma through the RPL5–MDM2–p53 pathway [6]. Ji Hoon Jung et al. found that MIDIIP1 promotes the progression of liver cancer through the co-localization of c-Myc mediated by ribosomal proteins L5 and L11 and CNOT2 [7]. Studies have also shown that MeCP2 can inhibit the transcription of RPL11 and RPL5 by binding to the promoter regions of RPL11 and RPL5, thereby promoting breast cancer cell proliferation and inhibiting apoptosis [8]. Some scholars have studied the antitumor effect of the antibacterial drug berberine and found that ribosomal protein (RP) L5 disappeared from the nucleolus and the accumulation of p53 protein in the nucleus in breast cancer MCF7 cells treated with 10 mM or 100 mM berberine, and RPL5 downregulation of berberine inhibited berberine-driven induction of p53 and p21 and cell death in MCF7 cells [16]. JiEonPark and other scholars have found that SanG can inhibit the proliferation of non-small cell lung cancer cells and induce their apoptosis through the combination of caspase-3 activation and RPL5-mediated c-Myc inhibition and doxorubicin in their research on the effect of Sangenong (SanG) [9]. The above research reports suggest that the role of RPL5 in tumors has potential value, but there is no report on the role and mechanism of RPL5 in colon cancer. In order to further clarify the role of RPL5 in colon cancer, it was found for the first time that RPL5 was highly expressed in colon cancer through bioinformatics database, clinical colon cancer tissue samples, and colon cancer cell line analysis, and downregulated RPL5 significantly inhibited the proliferation and migration ability of colon cancer cells.

The MAPK/ERK pathway is a key signaling pathway involved in the development of various cancers including CRC [11, 12]. Several studies have shown that the MAPK/ERK pathway is closely related to the pathogenesis of CRC [11, 12, 17, 18]. There are also genomic studies showing that the MAPK signaling pathway is one of the most frequently dysregulated pathways in CRC [19, 20]. As a key member of the zinc-dependent endopeptidase family, MMP9 has been widely considered to be involved in tumor cell proliferation and invasion [21, 22]. Further studies by Xu et al. showed that the use of ERK inhibitors reduced MMP expression and cell invasion and migration in CRC [18]. Therefore, further study on the role of RPL5 in MAPK/ERK pathway may help to reveal the potential molecular mechanism of RPL5 regulating COAD. In our study, RPL5 promoted the expression of p-MEK, p-ERK, c-Myc and inhibited the expression of FOXO3 in the MAPK/ERK pathway, which was partially reversed by the ERK activator TBHQ. Consistent with previous studies, the inhibition of MAPK/ERK was associated with decreased CRC cell proliferation and migration. Thus, we conclude that the promoting effect of RPL5 on colon cancer cell lines may be, at least in part, due to the activation of MAPK/ERK signaling.

As far as we know, previous studies on RPL5 in melanoma, liver cancer, breast cancer and non-small cell lung cancer have mentioned that RPL5 plays an important role in its occurrence and development, but none of them have been studied in depth. In our study, it was found that RPL5 is highly expressed in colon cancer, and more importantly, RPL5 regulates colon cancer proliferation and migration through the MAPK/ERK signaling pathway. This study suggests that RPL5 can be a potential therapeutic target for clinical treatment of colon cancer patients. However, we acknowledge several important limitations of this study. First of all, we did not evaluate the correlation between RPL5 expression and patient prognosis. Secondly, due to the small number of included patients, we were unable to evaluate the roles of RPL5 at different stages of COAD and the role of RPL5 in COAD metastasis. Considering the complex mechanisms underlying CRC development and endogenous biological differences in CRC cell lines, additional studies are needed.

## Conclusion

RPL5 promoted COAD cell proliferation and migration, at least in part, by activating the MAPK/ERK signaling pathway. These findings suggest that RPL5 exhibits an oncogenic effect in human COAD progression.

## Supplementary Information

**Additional file 1.**

## Data Availability

All of the data generated during this study were included in this article.
