# Supplementary material for: Ribosomal protein RPL5 regulates colon cancer cell proliferation and migration through MAPK/ERK signaling pathway
Source: BMC Mol Cell Biol. 2022 Nov 16;23:48. doi: 10.1186/s12860-022-00448-z (PMC9670436; doi:10.1186/s12860-022-00448-z)

Figure 2C

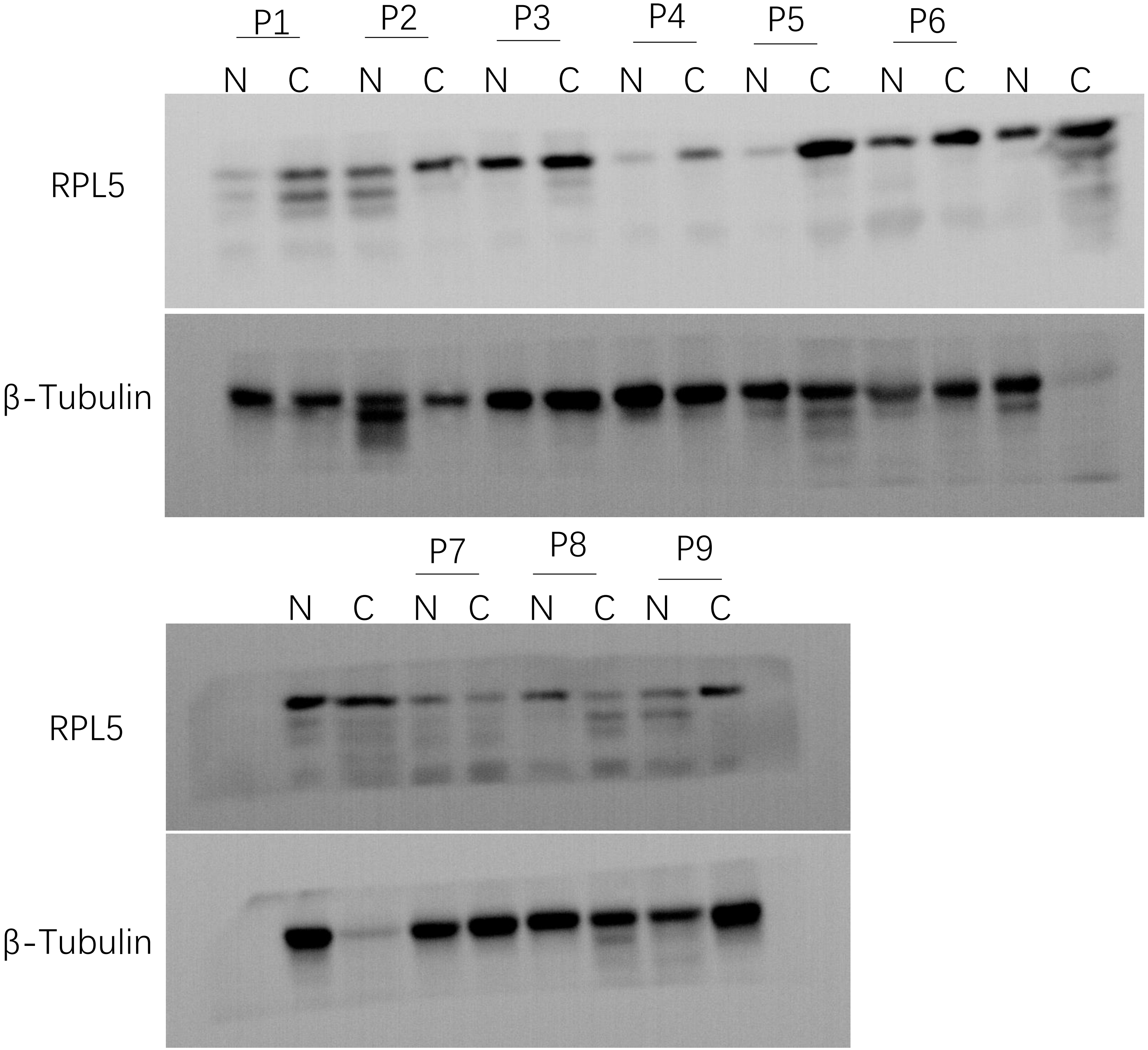
P1
P2
P3
P4
P5
P6
N C N C N C N C N C N C N C
RPL5
β-Tubulin
P7
P8
P9
N C N C N C N C
RPL5
β-Tubulin

Figure 2D

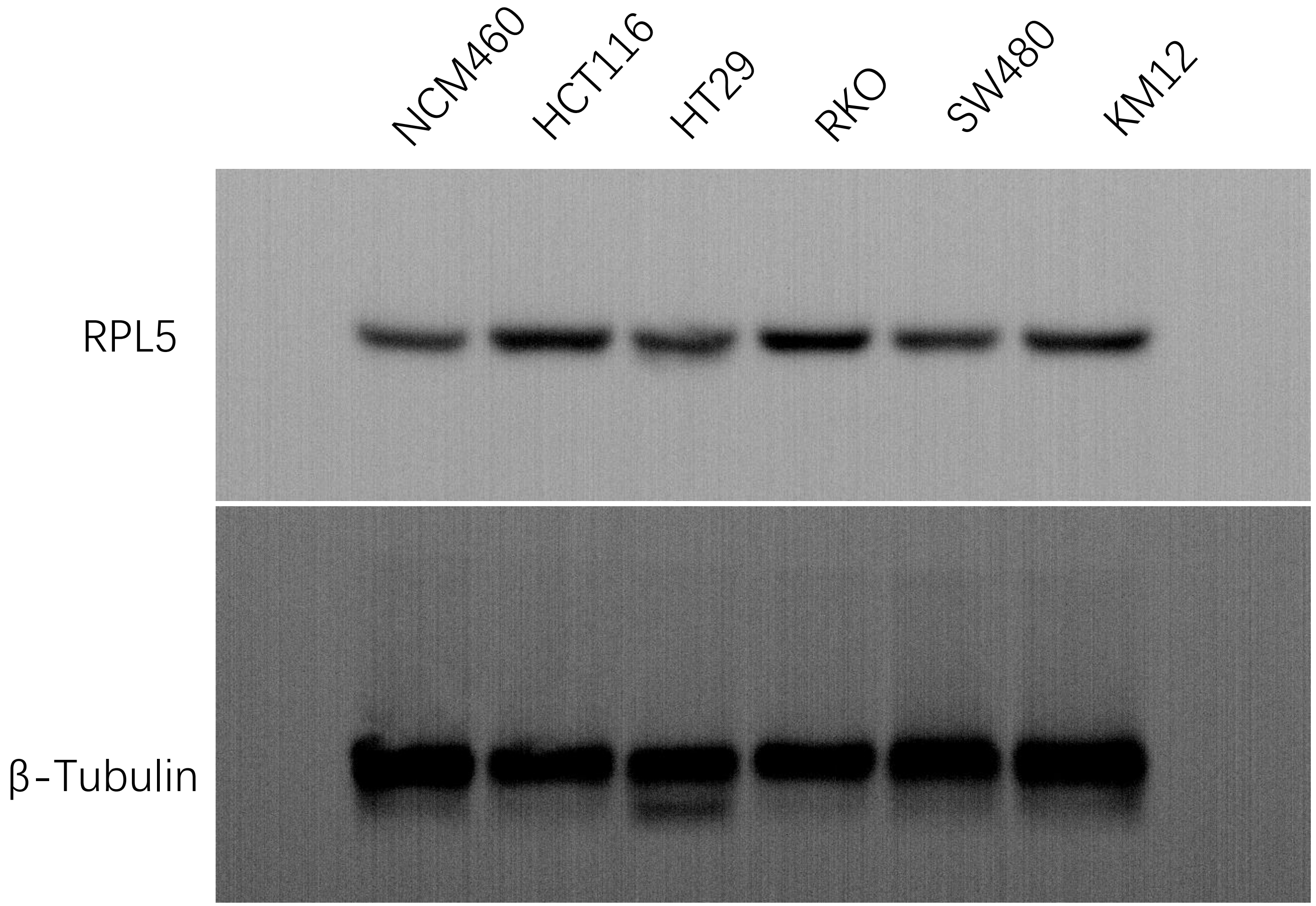

Figure 3A

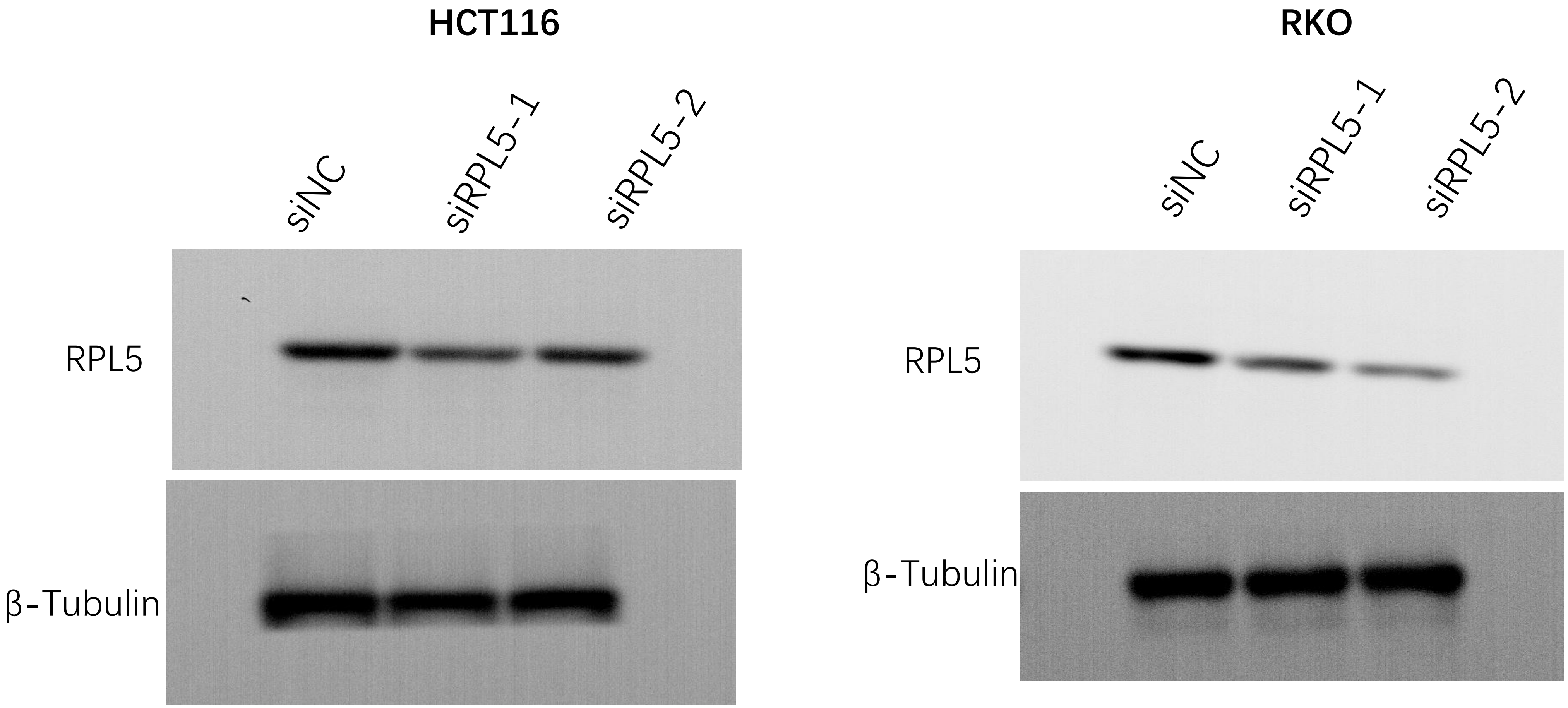
HCT116
RKO
siNC
siRPL5-1
siRPL5-2
siNC
siRPL5-1
siRPL5-2
RPL5
β-Tubulin
RPL5
β-Tubulin

Figure 5B

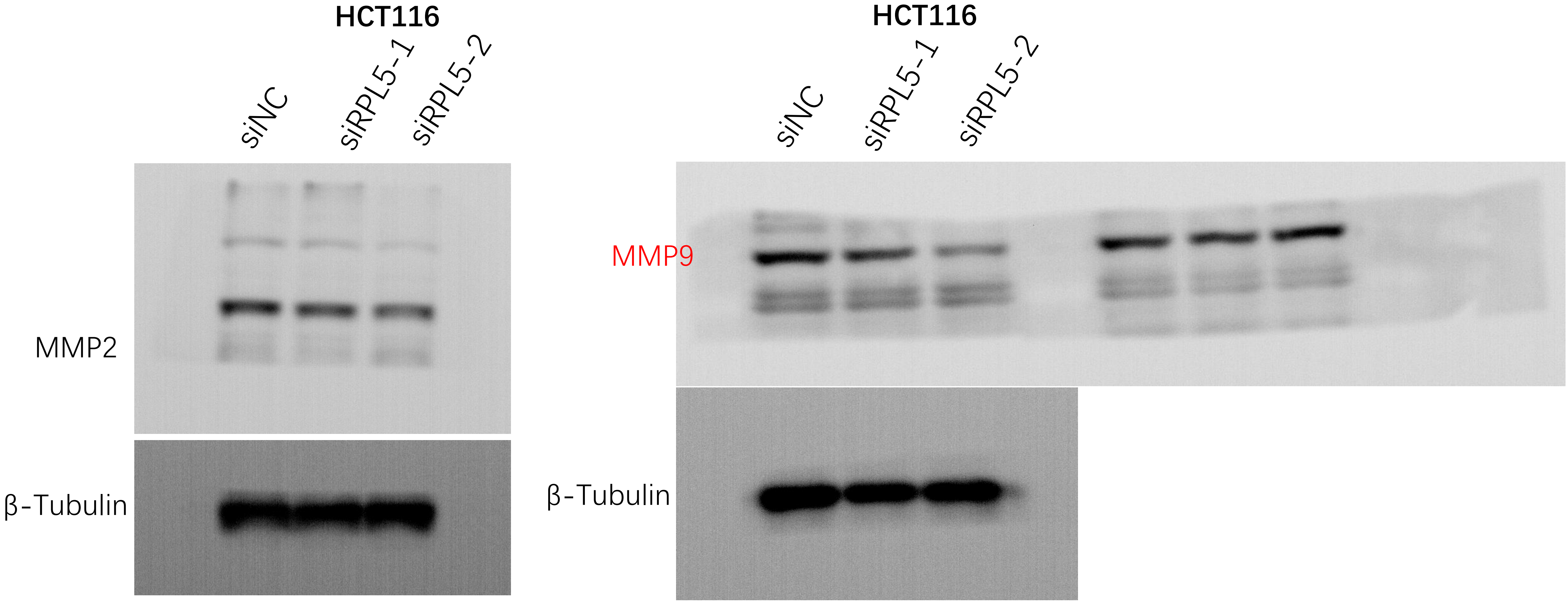
HCT116
siNC
siRPL5-1
siRPL5-2
MMP2
β-Tubulin
HCT116
siNC
siRPL5-1
siRPL5-2
MMP9
β-Tubulin

Figure 5B

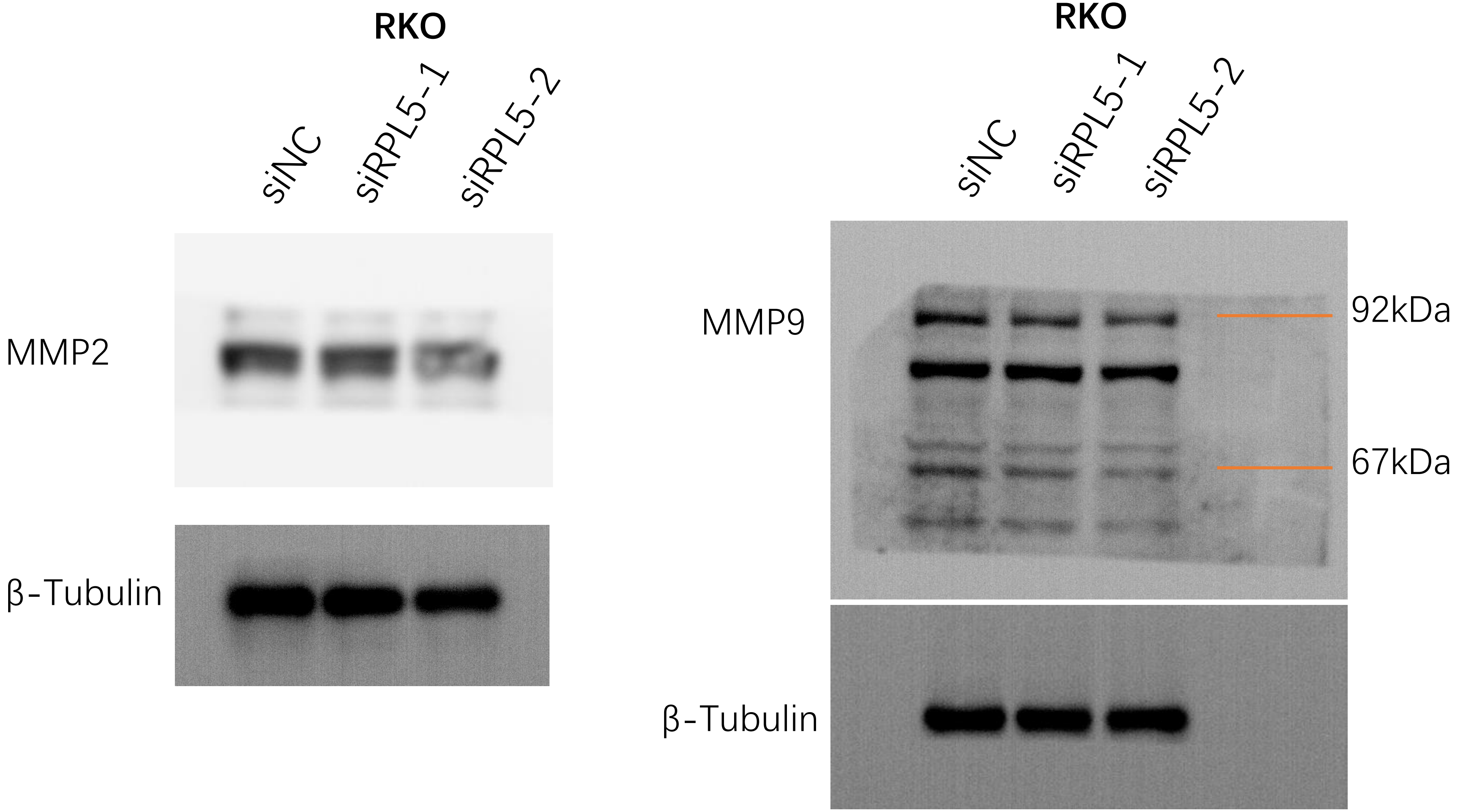
RKO
siNC
siRPL5-1
siRPL5-2
MMP2
β-Tubulin
RKO
siNC
siRPL5-1
siRPL5-2
MMP9
92kDa
67kDa
β-Tubulin

Figure 6B

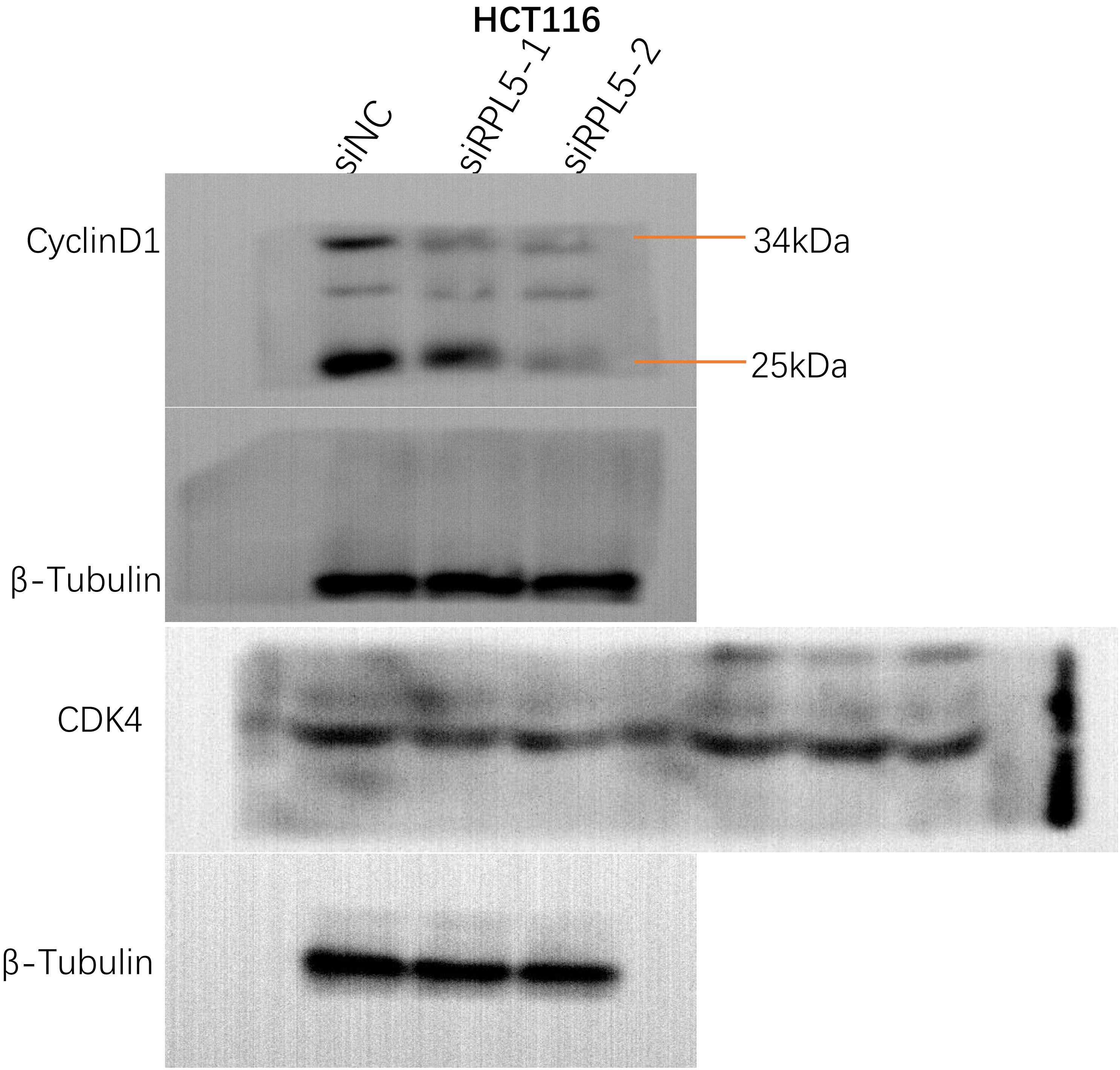
HCT116
siNC
siRPL5-1
siRPL5-2
CyclinD1
34kDa
25kDa
β-Tubulin
CDK4
β-Tubulin

Figure 6B

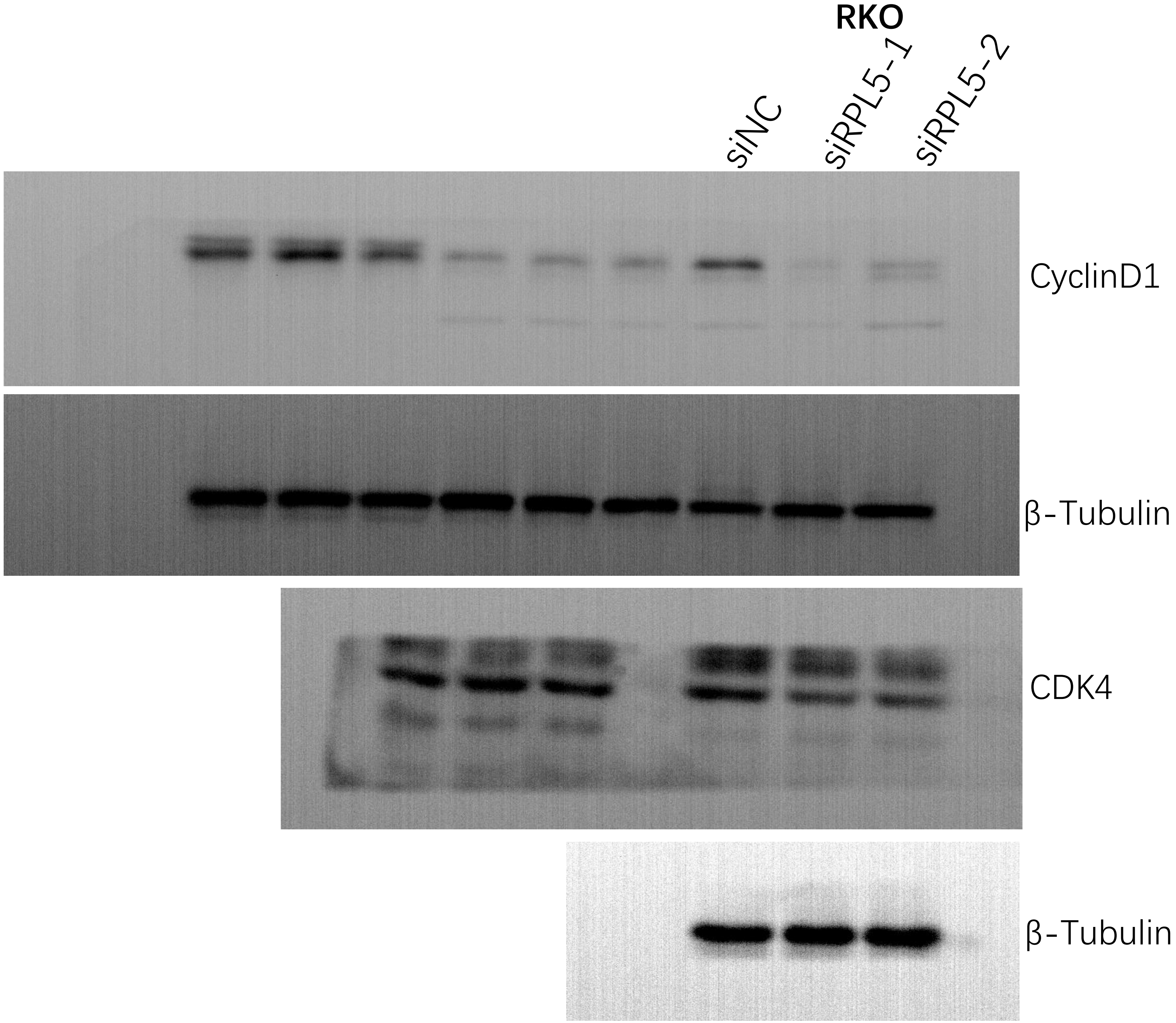

Figure 7A

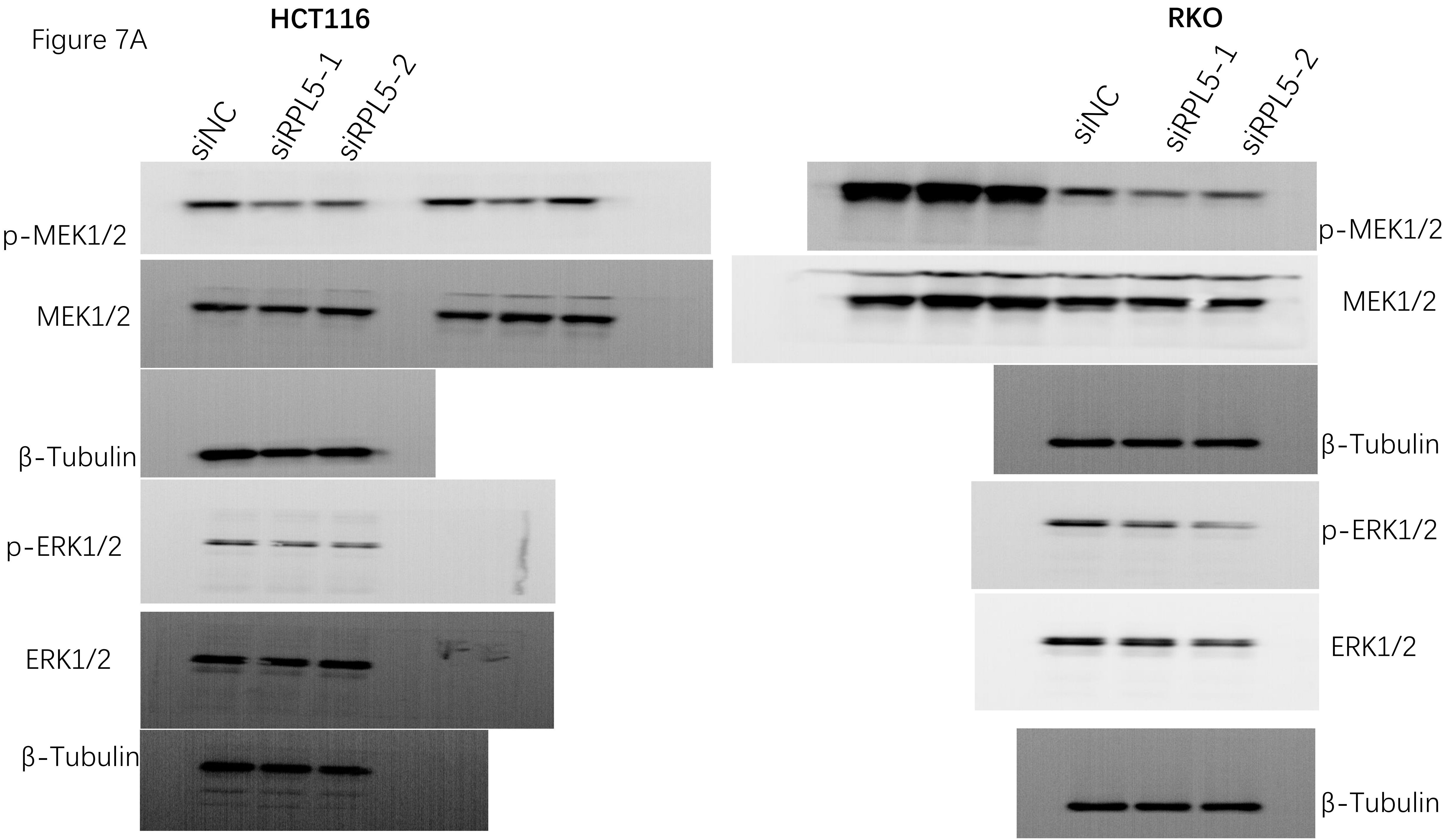
HCT116
siNC
siRPL5-1
siRPL5-2
p-MEK1/2
MEK1/2
β-Tubulin
p-ERK1/2
ERK1/2
β-Tubulin
RKO
siNC
siRPL5-1
siRPL5-2
p-MEK1/2
MEK1/2
β-Tubulin
p-ERK1/2
ERK1/2
β-Tubulin

Figure 7A

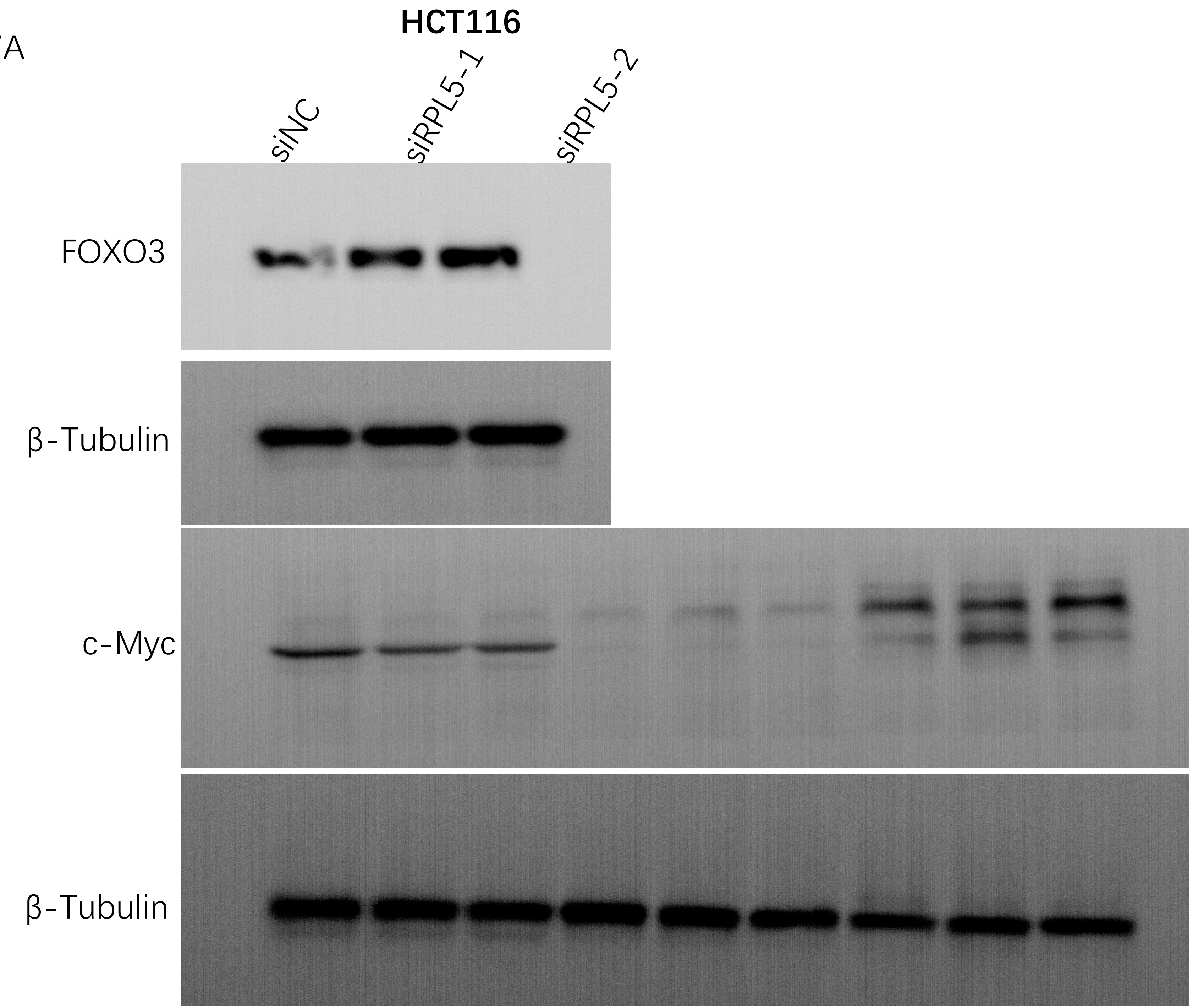

Figure 7A

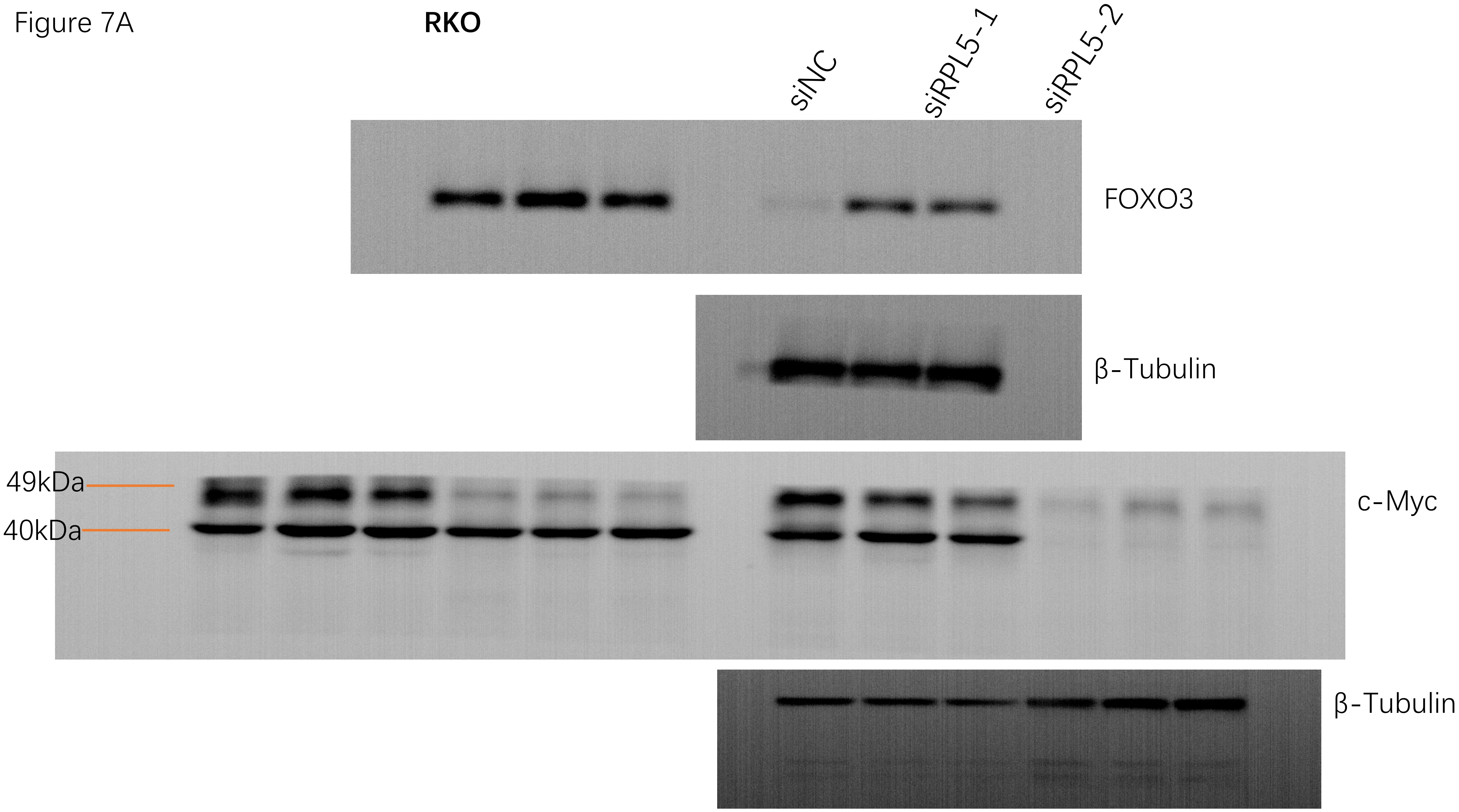
RKO
siNC
siRPL5-1
siRPL5-2
FOXO3
β-Tubulin
49kDa
40kDa
c-Myc
β-Tubulin

Figure 7B

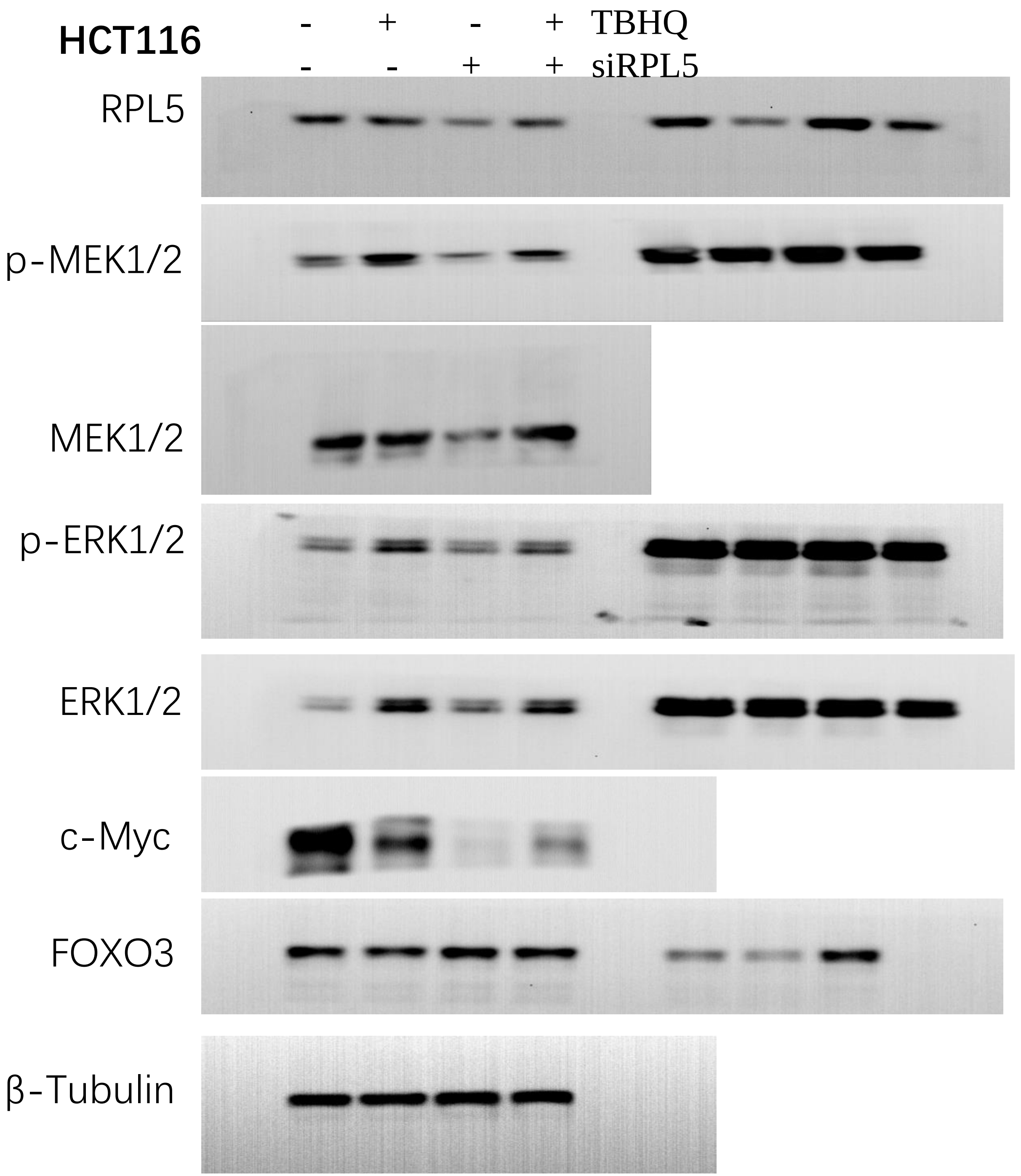
HCT116
- + - + TBHQ
- - + + siRPL5
RPL5
p-MEK1/2
MEK1/2
p-ERK1/2
ERK1/2
c-Myc
FOXO3
β-Tubulin

Figure 7B

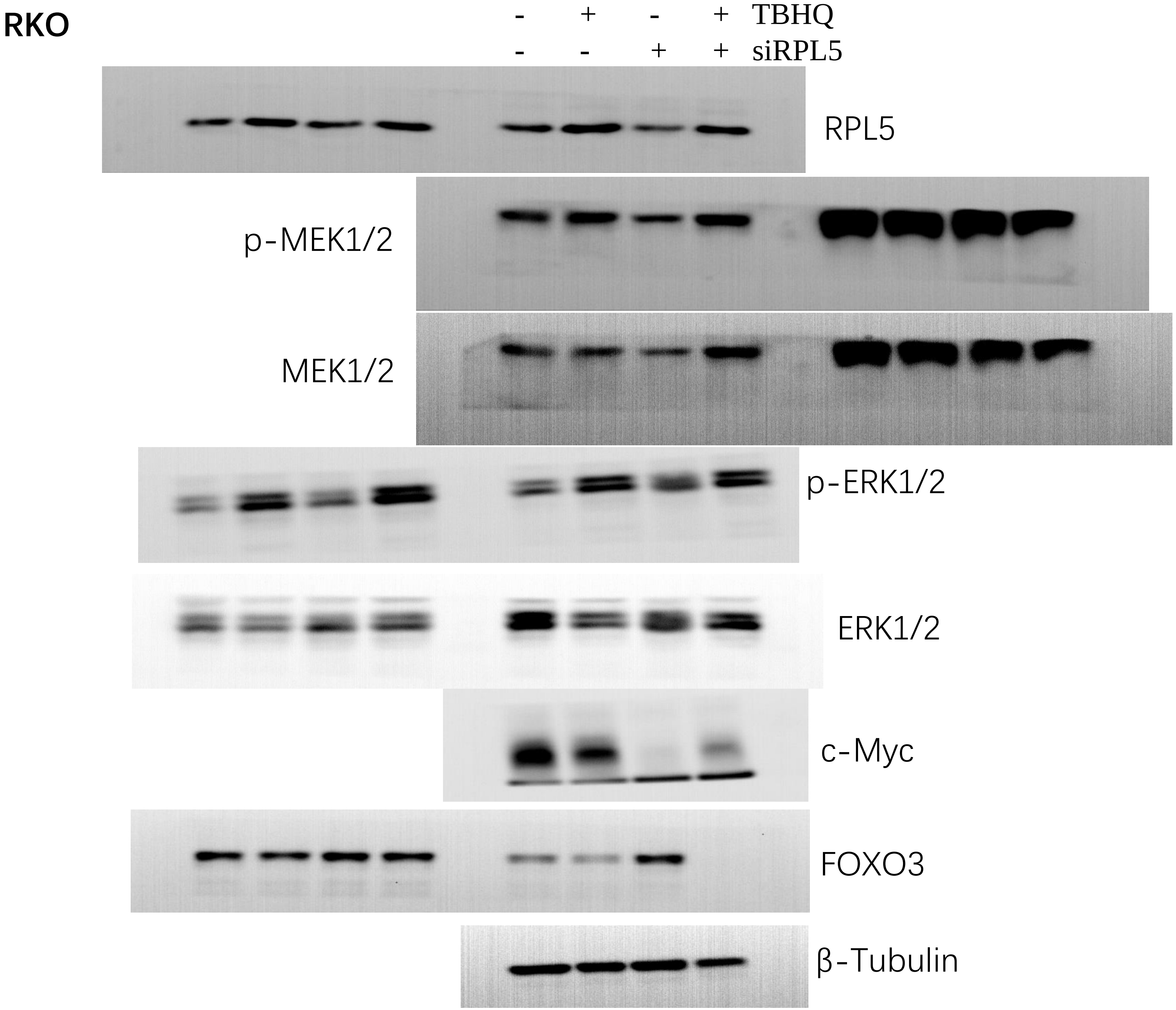
RKO
- + - + TBHQ
- - + + siRPL5
RPL5
p-MEK1/2
MEK1/2
p-ERK1/2
ERK1/2
c-Myc
FOXO3
β-Tubulin

Figure 7E

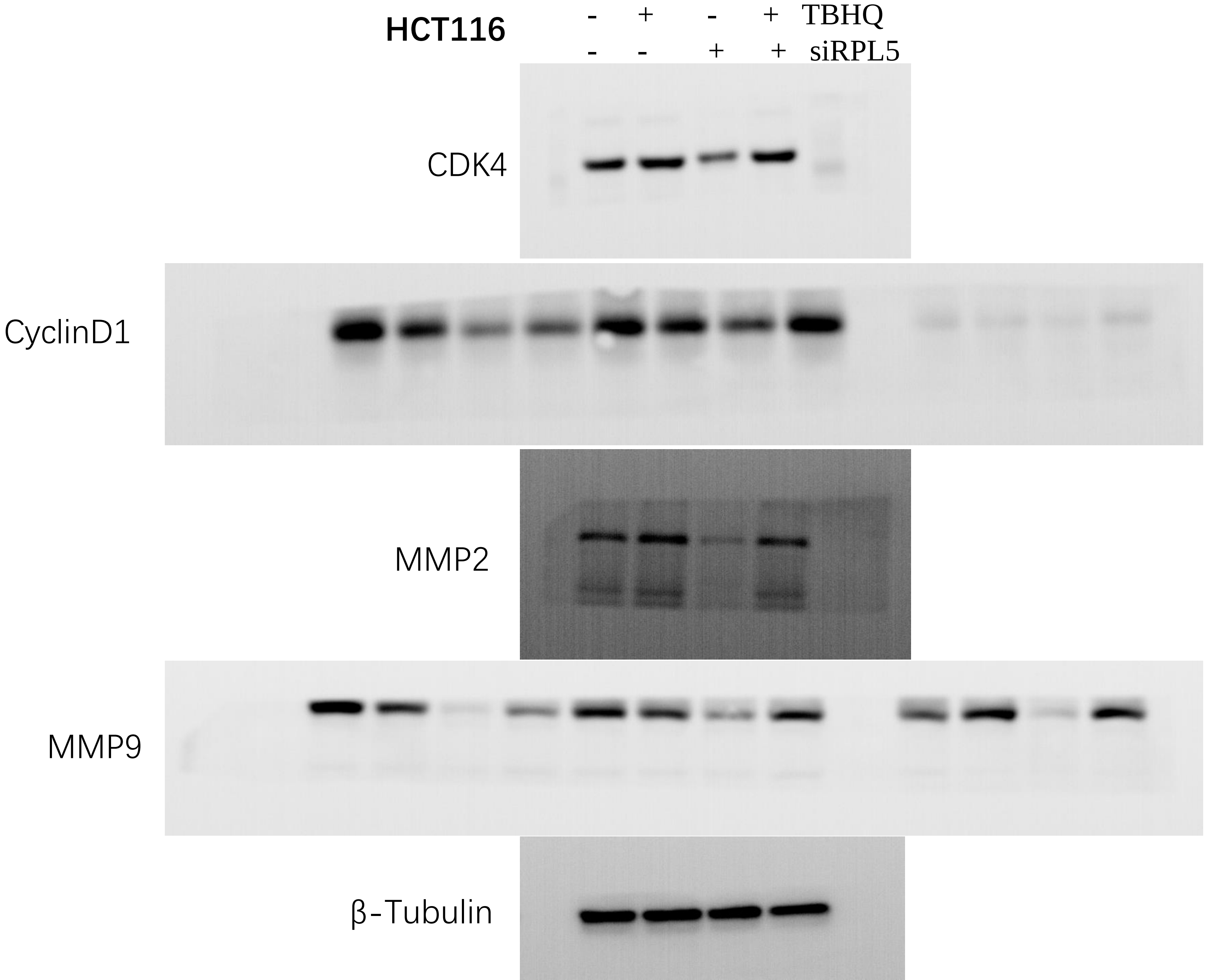

Figure 7E

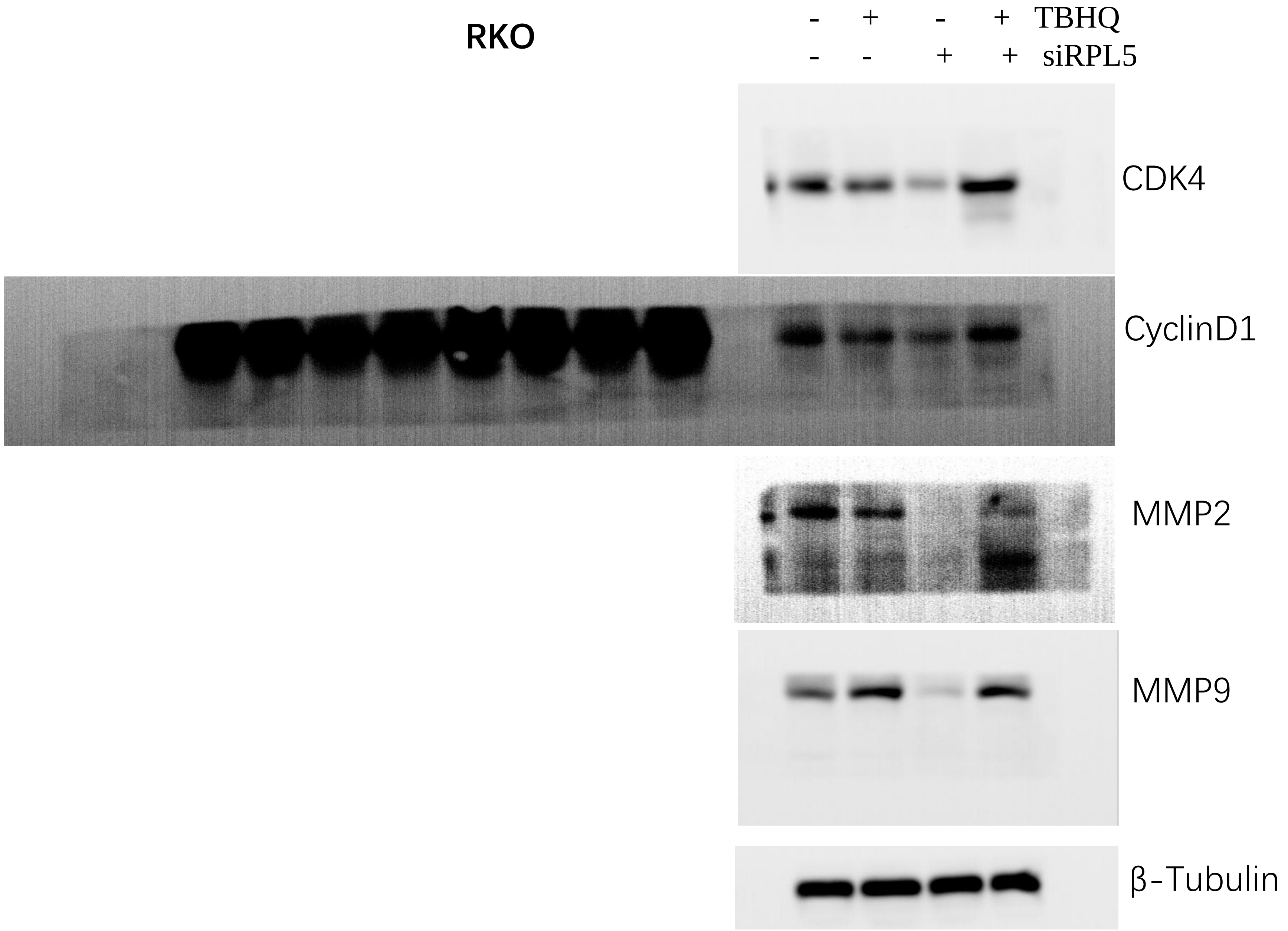

Supplement: Supplementary file 1 — Additional file 1. [file 12860_2022_448_MOESM1_ESM.pdf]
